# Are we prepared for potential repercussions of international sports events in the midst of a global pandemic?

**DOI:** 10.7189/jogh.13.03061

**Published:** 2023-12-27

**Authors:** Esteban Ortiz-Prado, Juan S Izquierdo-Condoy, Justin Yeager, Eduardo Vásconez-González

**Affiliations:** 1 One Health Research Group, Universidad de las Americas, Quito, Ecuador; 2 Biodiversidad Medio Ambiente y Salud (BIOMAS), Direccion General de Investigacion, Universidad de las Américas, Quito, Ecuador

## COMMENTARY

As we are now three years into a global pandemic, regional and international events are being held again. Such events feature spectators travelling from diverse geographic regions and grouping in high densities before returning to their homes. The recent 2022 Soccer World Cup held in Qatar brought together millions of people from all over the world, and thus provides us insights into how these large-scale international events may influence the future trajectory of the global coronavirus disease 2019 (COVID-19) pandemic. According to the Qatar airport authority data, between 34 to 36 million passengers have passed through Qatari airports during 2022 – double the numbers reported in 2021, which in November and December alone were estimated to be around 3.5 to 4.7 million, respectively [1].

**Figure 1:**
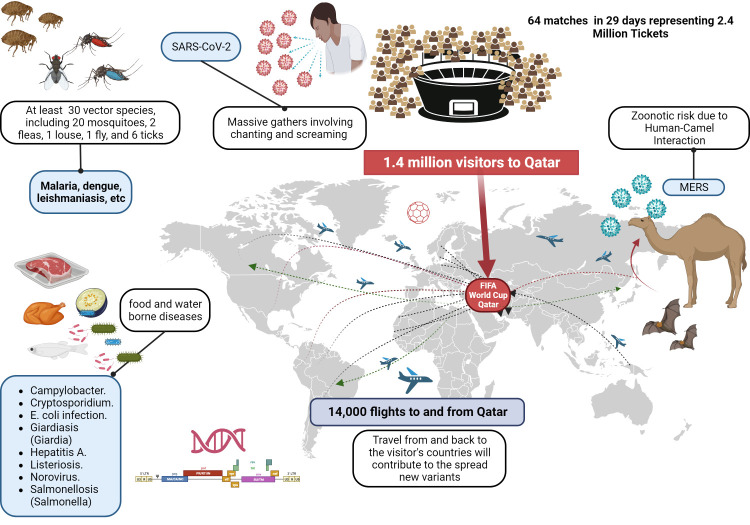
Infographic representing the main risks attributed to visiting a massive event, in this case the FIFA World Cup 2022 in Qatar. Created by the authors.

An international event of this magnitude brought together more than 1.4 million visitors, with an average attendance of 53 000 fans per match [2]. This, together with the measures decreed by the Qatari Ministry of Health, which mandated the use of masks inside health centers as the only personal protection measure [3], made physical distancing almost impossible throughout the event. Highly competitive sporting events intrinsically create ideal conditions for person-to-person transmission of various infectious diseases, which can spread rapidly worldwide due to today's ease of mobility [4]. Images of thousands of people in proximity to one another in subways, stadiums, restaurants, and squares; with such activities as singing out loud, cheering for their teams, hugging one another, shouting, laughing, and crying. With the same frequency as two teams meeting on the field, region-specific strains of coronaviruses (among others) could have been circulated within the crowd, leading to the potential for higher levels of global admixture. Likewise, there are justifiable concerns that novel emergent strains could similarly result (**Figure 1**).

As thousands of tourists took further advantage of their trip by booking tour packages during their visit to the Middle East, many visited the desert dunes, rode camels, and shared different cultures and customs with the locals. Notably, the camel beauty contest festival of the Camel Mzayen Club was held simultaneously with the World Cup [5], allowing thousands of people to interact closely with camels which are known carriers of zoonotic diseases. This interaction inadvertently created conditions conducive to the transmission of camel-associated pathogens, including the Middle East respiratory syndrome (MERS), a disease transmitted through direct or indirect contact with camels. Although the mechanism of transmission has not been fully described, routes such as urine contact and the consumption of raw dairy products or meat have been implicated as means of MERS transmission [6]. Similarly, the heightened risk of vector-borne diseases cannot be ignored, as many travellers had never been in contact with these mammals before, potentially amplifying the risk [7].

At the end of the Qatar 2022 World Cup, hundreds of new cases of aerosol-borne diseases were reported in Qatar; in fact, the country reported an average of 321 cases daily in November 2022 [8], while the evolution in the transmission of confirmed COVID-19 cases since the opening of the World Cup showed a very close correlation with the arrival of fans, as determined by an increase in cases that peaked (more than 100% increase in confirmed cases) during the middle of the World Cup (5 December) and gradually decreased as it approached its final stages [9]. Meanwhile, a large, but undetermined number of tourists complained of undiagnosed respiratory illnesses, and several professional footballers were forced to rest and isolated after being diagnosed with what approximated the MERS [10]. Although the diagnosis of these cases was never officially confirmed, in scenarios involving potentially transmissible zoonotic diseases such as Qatar – which, along with Saudi Arabia, has reported 80% of MERS cases in humans – and in the case of highly serious diseases such as MERS (fatality rate = 35%), an environment with hundreds of thousands of people from around the planet close to natural reservoirs of the disease )as is the case with camels) poses an exceedingly high risk to global health [11].

Now that the World Cup is over, as thousands of fans and tourists return to their home countries, they have surely met with friends and family to share their experiences. Given the uncertainty surrounding whether the risk abated after the conclusion of the World Cup or if this globally significant event directly contributed to the rise in respiratory disease cases, as well as whether the impact of shortcomings in control measures will once again manifest in global health systems, it is evident that a complex interplay of factors is at play. An illustrative example is found in Argentina, where history witnessed one of the most monumental celebrations on 18 December 2022, following their triumph in the Qatar 2022 World Cup. Local authorities reported an astounding gathering of at least five million individuals occupying the streets, consequently presenting a considerable avenue for the transmission of infectious diseases. This manifested in a discernible increase in the recorded case count during the initial week of January 2023, with official records indicating 72 558 reported infections [12], underlining the impact of mass gatherings on disease transmission dynamics.

By the end of 2022, the excessive increase in cases observed in Qatar joined countries such as China and Japan, which have been affected by COVID-19 to an unprecedented extent [13,14]. These scenarios clearly demonstrated that the pandemic caused by severe acute respiratory syndrome coronavirus 2 (SARS-CoV-2) has not been overcome and that the control strategies established in the world have left aside a scenario that has not yet been overcome. However, while we are aware that large international events such as World Cups, carnivals, year-end festivals and others can bring together large groups of people – are we truly prepared in our pre-planning and monitoring? To this end, responsible conduct must accompany the authorities’ active participation in event planning, which minimally should include a return to mandatory personal protection measures such as the use of masks in closed spaces, control and monitoring of new cases through laboratory tests, genomic characterisation of viruses, and the implementation of vaccination campaigns for potentially transmissible diseases before the event. We fully understand that the return of the population to activities and events that took place in pre-pandemic conditions is fundamental; however, we also believe that, together with participants’ responsible conduct, authority-mandated regulations are essential for controlling the spread of infectious diseases such as COVID-19.

With more international events planned in the near or farther future, such as the Olympic Games, the Union of European Football Associations (UEFA) Champions League, the Cricket World Cup, or the Super Bowl, we highlight a need for proper reflections; lessons learned immediately after the World Cup could be applied to mitigate further unnecessary regional complications to global problems.

We advocate for the establishment of a stringent global surveillance agenda to oversee and manage such large-scale events. This initiative would involve the collaboration of an international team of experts, capable of deployment to the host country. Their role would encompass understanding the local social and cultural context, as well as integrating with the local planning team responsible for orchestrating these events. This cooperative approach could tailor measures that align with the specific scenario, thereby mitigating the risk of zoonotic diseases and emerging or resurging illnesses, and preventing their transmission to travellers’ countries of origin post-event.

Furthermore, it is imperative to recognise that, given the infrequent occurrence of mass gatherings like the *Fédération internationale de football association* (FIFA) World Cup or the Olympics, bolstering national and international communication channels among public health and epidemiological surveillance personnel is of paramount importance. This strengthened communication network would serve as a formidable defence against the potential propagation of infectious diseases that could otherwise stem from these events' expansive reach.
